# First recording of cardiac activity in the *Anteos menippe* bubble and isoflurane interference during anesthetic induction and recovery

**DOI:** 10.1007/s00360-026-01684-x

**Published:** 2026-07-27

**Authors:** Antônio Basílio Guerreiro Júnior, Deise de Lima Cardoso, Axell Lins, Sabrina Reika Seko Kondo, Naoto da Cunha Hayasaki, Eduardo Machado da Silva Zamian, Alexa Camila Lopes de Assis, Diogo Macola Felix, Eva Vitória Ferreira Viana, Diva Anelie de Araújo Guimarães, Nilton Akio Muto, Moisés Hamoy

**Affiliations:** 1 Laboratory of Pharmacology and Toxicology of Natural Products, Institute of Biological Sciences, Federal University of Pará (ICB-UFPA), R. Augusto Corrêa, 01, Guamá, Belém, 66075-110 Pará Brazil; 2 Department of Veterinary Medicine, Mangal das Garças Zoo-Botanical Park, Belém, 66020-160 Pará Brazil; 3 Animal Reproduction Laboratory, Institute of Biological Sciences, Federal University of Pará (ICB-UFPA), Belém, CEP: 66075-900 Pará Brazil; 4 Center for the Valorization of Bioactive Compounds from the Amazon, Guamá Science and Technology Park (PCT-Guamá), Federal University of Pará (UFPA), Belém, Pará Brazil

**Keywords:** *Anteos menippe*, Isoflurane, Electrocardiography, Cardiac physiology, Autonomic modulation, Invertebrate anesthesia

## Abstract

Understanding the physiological regulation of cardiac activity in insects remains a central topic in insect biochemistry and physiology. In Lepidoptera, direct electrophysiological characterization of cardiac function is still limited, particularly under anesthetic conditions commonly used during experimental manipulation. In this study, we evaluated the effects of isoflurane anesthesia on cardiac electrophysiology in adult *Anteos menippe* butterflies during anesthetic induction and recovery. A total of eighteen newly emerged adults were subjected to two experiments. Experiment 1 - behavior during anesthetic induction and recovery with isoflurane (*n* = 6). Experiment 2 - the animals were divided into a control group (*n* = 6) and a group treated with isoflurane (*n* = 6). The animals were monitored during anesthetic induction and recovery with isoflurane; in this case, electrocardiographic signals (ECG) were obtained using 0.06 mm diameter tungsten steel alloy electrodes, implanted to acquire cardiac recordings lasting 30 min under controlled conditions. Isoflurane induced mild and reversible anesthesia, with induction and recovery times of 48.5 ± 8.9 s and 117.7 ± 16.2 s, respectively, without observable excitatory responses. During anesthesia, the frequency of cardiac action potentials decreased significantly, followed by a gradual recovery accompanied by increases in amplitude and linear signal power. Spectral analysis revealed a dynamic redistribution of energy during anesthetic elimination, consistent with chronotropic modulation of cardiac activity. These findings provide new electrophysiological insights into the cardiac physiology of butterflies and support the use of isoflurane as a reliable anesthetic for experimental studies involving cardiac monitoring in lepidoptera and other insects.

## Introduction

Butterflies are highly sensitive insects widely recognized as ecological bioindicators due to their rapid physiological responses to environmental disturbances, habitat fragmentation, climate change, and chemical contamination (Dessuy and Morais 2007). In addition to their ecological importance as pollinators, Lepidoptera species have increasingly been used in conservation programs, captive breeding initiatives, ecotoxicological monitoring, and physiological studies. Because of their environmental sensitivity, the establishment of safe handling and immobilization procedures is essential to minimize stress and ensure animal welfare during laboratory manipulation, transport, physiological monitoring, and experimental procedures.

The order Lepidoptera, which includes butterflies and moths, presents high diversity in Brazil and plays a fundamental role in ecosystem maintenance (Prado and Zukovski 2012). Among these insects, the Pieridae family includes migratory butterflies distributed mainly in tropical and temperate regions of South America (Monteiro et al. 2009). *Anteos menippe*, belonging to the subfamily Coliadinae, is characterized by predominantly yellow and orange coloration and represents an ecologically relevant species within this group (Born and Lima, 2005; Monteiro et al. 2009). Due to the environmental fragility of butterflies, several management practices are necessary to reduce physiological stress during handling, transport, and experimental manipulation.

In this context, anesthesia in invertebrates has become an important tool for facilitating handling procedures, physiological recordings, biological sampling, imaging analyses, and transport while reducing stress and minimizing physical damage (Cooper 2001). Traditionally, immobilization in insects has been performed using cold exposure or carbon dioxide (CO₂) anesthesia (Nilson et al. 2006). However, previous studies have demonstrated that these methods may induce physiological disturbances, including metabolic suppression, delayed recovery, alterations in ion balance, and changes in cardiac and neural activity (Gooley and Gooley 2021). Although butterflies generally exhibit lower metabolic demands than highly active insects such as bees, the physiological consequences of cold and CO₂ exposure in Lepidoptera remain poorly understood, particularly regarding cardiac function and electrophysiological regulation.

Therefore, volatile anesthetics such as isoflurane emerge as promising alternatives for insect management. Isoflurane (2-chloro-2-(difluoromethoxy)−1,1,1-trifluoroethane) primarily acts as a positive modulator of GABA-A receptors, increasing chloride ion permeability and inducing neuronal hyperpolarization. These mechanisms promote general anesthesia with hypnotic, sedative, and muscle-relaxant effects. In insects, the GABAergic system is highly conserved and plays a central role in regulating motor activity and neural excitability (Buckingham et al. 2005). Isoflurane has demonstrated rapid induction, predictable recovery, and reduced long-term physiological stress in several invertebrate species, including bees, mantises, and scorpions (MacMillan et al. 2017; D’Ovidio et al. 2021; Gaudette et al. 2022; Contrera et al. 2023). Nevertheless, studies specifically evaluating anesthetic effects in butterflies remain extremely limited.

Butterflies are also valuable organisms for ecotoxicological and physiological investigations because environmental stressors and chemical contaminants may directly affect survival, migration, reproduction, pollination efficiency, metabolism, and cardiovascular regulation. In this context, electrophysiological approaches may provide highly sensitive biomarkers for detecting physiological alterations induced by stress or toxic exposure. However, despite advances in insect neurophysiology, direct electrophysiological characterization of cardiac activity in Lepidoptera remains scarce.

Electrocardiographic (ECG) monitoring has emerged as a useful tool for investigating cardiac dynamics, chronotropic modulation, and physiological stress responses in invertebrates. Although the principles of ECG acquisition and signal analysis are well established in vertebrate models (Berkaya et al. 2018), their application in insects is directed toward the characterization of electrical activity patterns rather than direct physiological comparisons with vertebrate cardiac systems. In insects, cardiac activity is regulated by a complex interaction between myogenic pacemaker activity and neurogenic modulation, allowing rapid physiological adjustments to internal and external stimuli (Sláma 2012). Despite this functional complexity, most studies involving insect cardiac physiology still rely on indirect measurements of hemolymph flow or optical approaches, which provide limited resolution of electrical conduction dynamics and autonomic modulation (Davis and Orakzai 2016).

Although vertebrate studies, such as those performed in mice (Berul et al. 1996), established important methodological foundations for electrophysiological recordings, the anatomical organization and regulatory mechanisms of insect cardiac systems differ substantially from those of vertebrates. Therefore, vertebrate studies are cited here exclusively as methodological references and not as direct physiological analogs. In this context, ECG recordings in insects may provide important information regarding cardiac rhythmicity, stress responses, metabolic regulation, and anesthetic effects, expanding the understanding of physiological adaptations and evolutionary conservation of neural control pathways across taxa (MacMillan et al. 2017; Wang and Chahl 2025).

Furthermore, obtaining stable electrophysiological recordings in butterflies remains technically challenging because movement artifacts and handling stress may interfere with signal acquisition and reproducibility. Consequently, the establishment of anesthetic protocols capable of promoting rapid, reversible, and physiologically stable immobilization is essential for improving electrophysiological investigations in Lepidoptera. Despite the growing interest in insect physiology and welfare, standardized ECG methodologies associated with anesthesia protocols for butterflies are still lacking.

Therefore, this study aimed to evaluate the use of isoflurane anesthesia in *Anteos menippe* butterflies by describing behavioral responses during anesthetic induction and recovery and characterizing cardiac electrophysiological activity throughout these processes. In addition, this study sought to establish an experimental framework for ECG monitoring in butterflies, contributing to future physiological, ecotoxicological, and comparative investigations involving Lepidoptera and other invertebrates.

## Materials and methods

### Animals

This study used eighteen newly emerged adult Anteos menippe butterflies (males and females) obtained from a breeding facility located in the Mangal das Garças Environmental Park, Belém, Pará, Brazil. The butterflies were transported to the Laboratory of Pharmacology and Toxicology of Natural Products of the Institute of Biological Sciences of the Federal University of Pará (ICB-UFPA), in an environment with controlled temperature (25 ± 0.3 °C).

Before the experimental procedures, the butterflies were acclimated to laboratory conditions for a stabilization period under controlled temperature (25 ± 0.3 °C) and minimal environmental disturbance. During acclimation, the animals were maintained under natural photoperiod conditions and monitored to ensure normal posture, wing movement, and behavioral activity prior to anesthesia and electrophysiological recording. This procedure was performed to minimize stress-induced physiological variations and stabilize cardiac activity before ECG acquisition.

### Behavioral analysis

**Fig. 1:**
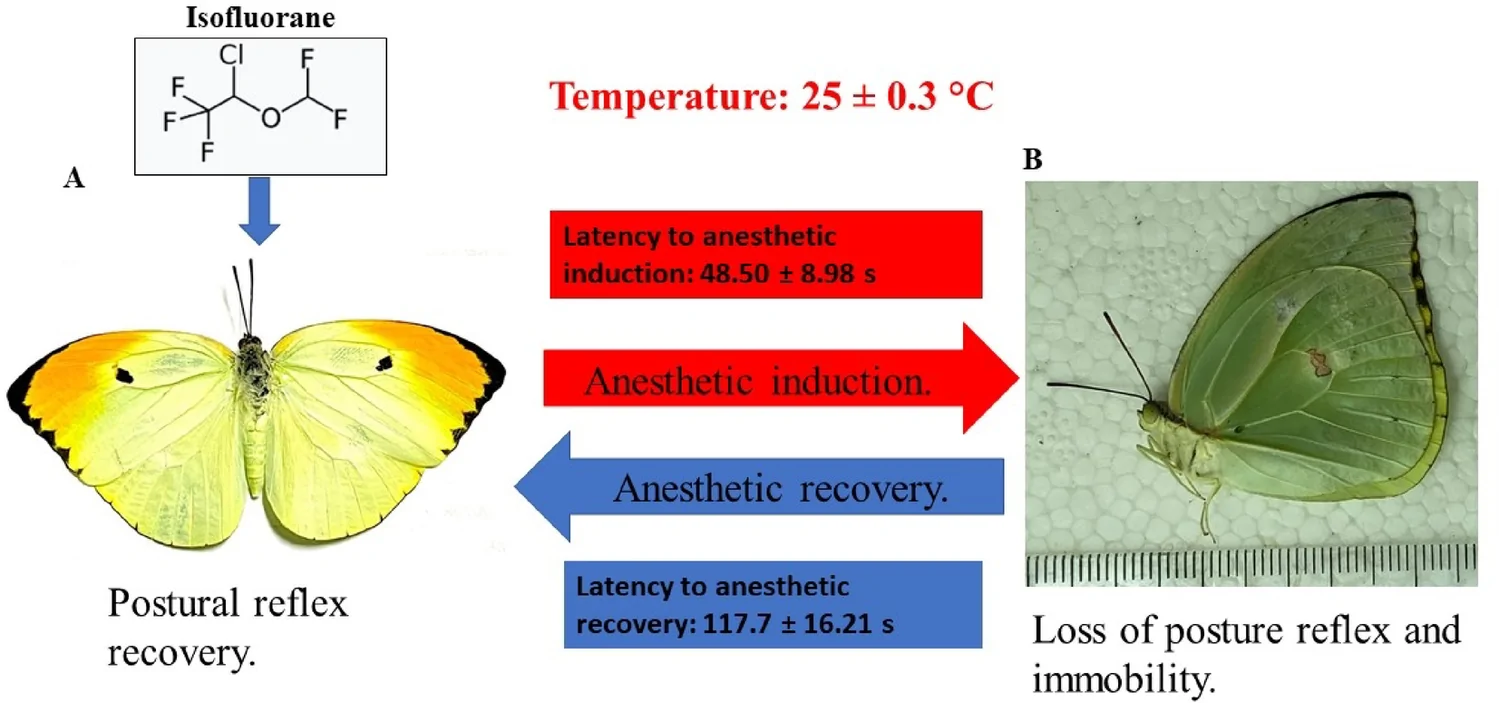
Behavioral characteristics of *Anteos menippe* butterflies during anesthetic induction and recovery with isoflurane: butterfly with normal postural reflex (**A**), butterfly with loss of postural reflex and immobility (**B**)

To analyze the latency of induction and recovery from isoflurane anesthesia, six butterflies were initially anesthetized with isoflurane. Isoflurane was selected due to its rapid induction and recovery characteristics, as well as its lower potential to induce prolonged physiological stress compared to traditional immobilization methods such as cold exposure or CO₂ anestesia, using a 1 mL volume impregnated in cotton and placed inside an anesthetic chamber with a capacity of 1500 cm³ at 25 ± 0.3 °C. The latency for loss of postural reflex and immobility was observed (Fig. 1B), and subsequently, during the recovery period, the latency and recovery of the postural reflex of the butterflies were observed (Fig. 1A).

### Electrode fabrication, implantation, and ECG recording

For electrocardiographic (ECG) recording, electrodes were constructed from JST SM cables with a 2-pin connector and a length of 13 cm. The paired electrodes were spaced 1 mm apart and constructed from tungsten alloy steel wire (B&R brand) with a diameter of 0.06 mm and a length of 2 mm (Fig. 3C). The electrodes were insulated with liquid insulator. The method of Contrera et al. (2023) was adapted with modifications, according to the equipment and materials available in this study.

**Fig. 2:**
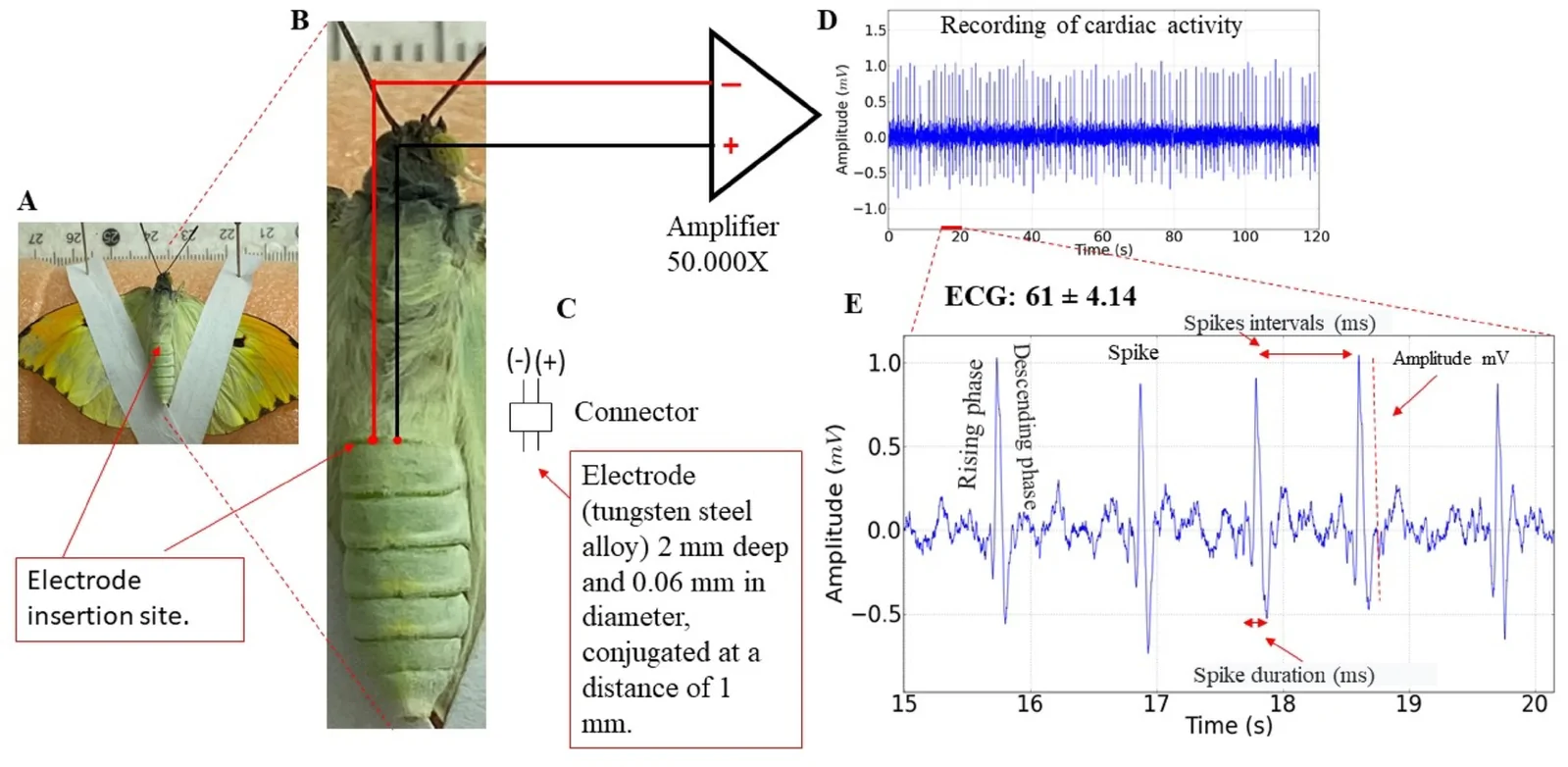
Preparation for ECG recording in *Anteos menippe* butterflies. Butterfly restraint for electrode positioning (**A**), magnified view showing the first abdominal spiracle and the positions of the recording and reference electrodes (**B**), model of the electrode used and its characteristics for implantation (**C**), representative cardiac recording of *Anteos menippe* (**D**), 5-second segment of the recording and a 0.12-second amplified trace of the ECG showing the ascending and descending phases of the spike, spike frequency per minute, spike amplitude (mV), interspike interval (ms), and spike duration (ms) (indicated by red arrows) (**E**). Signal amplification: 50,000×; characteristic signal observed on the monitor

The butterflies were restrained using paper strips and pins (Fig. 2A and B). After fixation, the electrodes were positioned according to the following coordinates: along the median sagittal line of the dorsal surface, adjacent to the first abdominal spiracle after the thoracic region, with a lateral displacement of ± 1 mm and a dorsoventral depth of 2 mm (Fig. 2B and C). The recording electrode was positioned on the right side and the reference electrode on the left. After electrode implantation, electrocardiograms were recorded (Fig. 2D and E).

For the electrophysiology experiment, butterflies were randomly divided into two groups: (a) control group (without isoflurane anesthesia) (*n* = 6) and (b) group treated with isoflurane under anesthesia for 5 min (*n* = 6). For the treated group, 1 mL of isoflurane was impregnated into cotton and placed inside an anesthetic chamber with a capacity of 1500 cm³ at 25 ± 0.3 °C, with anesthesia lasting 5 min. Subsequently, the isoflurane was removed to allow for anesthetic recovery of the butterflies. All recordings lasted 30 min.

All recordings were performed during the same period of the day in order to minimize possible circadian influences on cardiac act.

All procedures were performed inside a Faraday cage with metallic shielding. The electrodes were connected to a high-impedance amplifier (Grass Technologies P511, West Warwick, USA) with a signal amplification of 50,000×, monitored by an oscilloscope (Protek, 6510). Each butterfly was recorded for a duration of 30 min. The animals remained in contact with isoflurane for 5 min for anesthetic induction, and recovery from anesthesia was subsequently observed and monitored by ECG during the remaining 25 min. Data were analyzed every 5 min of recording, as follows: 0–300 s (A), 300–600 s (B), 600–900 s (C), 900–1200 s (D), 1200–1500 s (E), and 1500–1800 (F). The recordings allowed evaluating the following parameters: peak frequency per minute, peak amplitude, inter-peak interval, peak duration, and signal power.

### Statistical analysis

The homogeneity of variances between groups was tested using Levene’s tests, using the Python program through the Scipy library. Data are presented as mean ± standard deviation (SD). A significance level of *p* < 0.05, *p* < 0.01, and *p* < 0.001 was considered for all analyses. Comparisons between periods were performed using one-way ANOVA followed by Tukey’s post hoc test for multiple comparisons. Statistical analyses were carried out using GraphPad Prism, version 8 (GraphPad Software Inc., San Diego, CA, USA). Data normality was verified using the Shapiro–Wilk test prior to parametric analyses.

## Results

Behavioral testing demonstrated that during anesthetic induction with isoflurane at 25 ± 0.3 °C, a gradual deepening of anesthesia was observed, characterized by loss of postural reflex and immobility, with a latency of 48.50 ± 8.98 s, (CV: 18.52%)(Fig. 1A). Anesthetic recovery occurred after removal of the butterfly needles from the isoflurane chamber, with the return of the postural reflex observed after 117.7 ± 16.21s (CV:13.77%). No excitability-related behavior was detected during the recovery period, and anesthesia relief occurred gradually and smoothly (Fig. 1B).

**Fig. 3:**
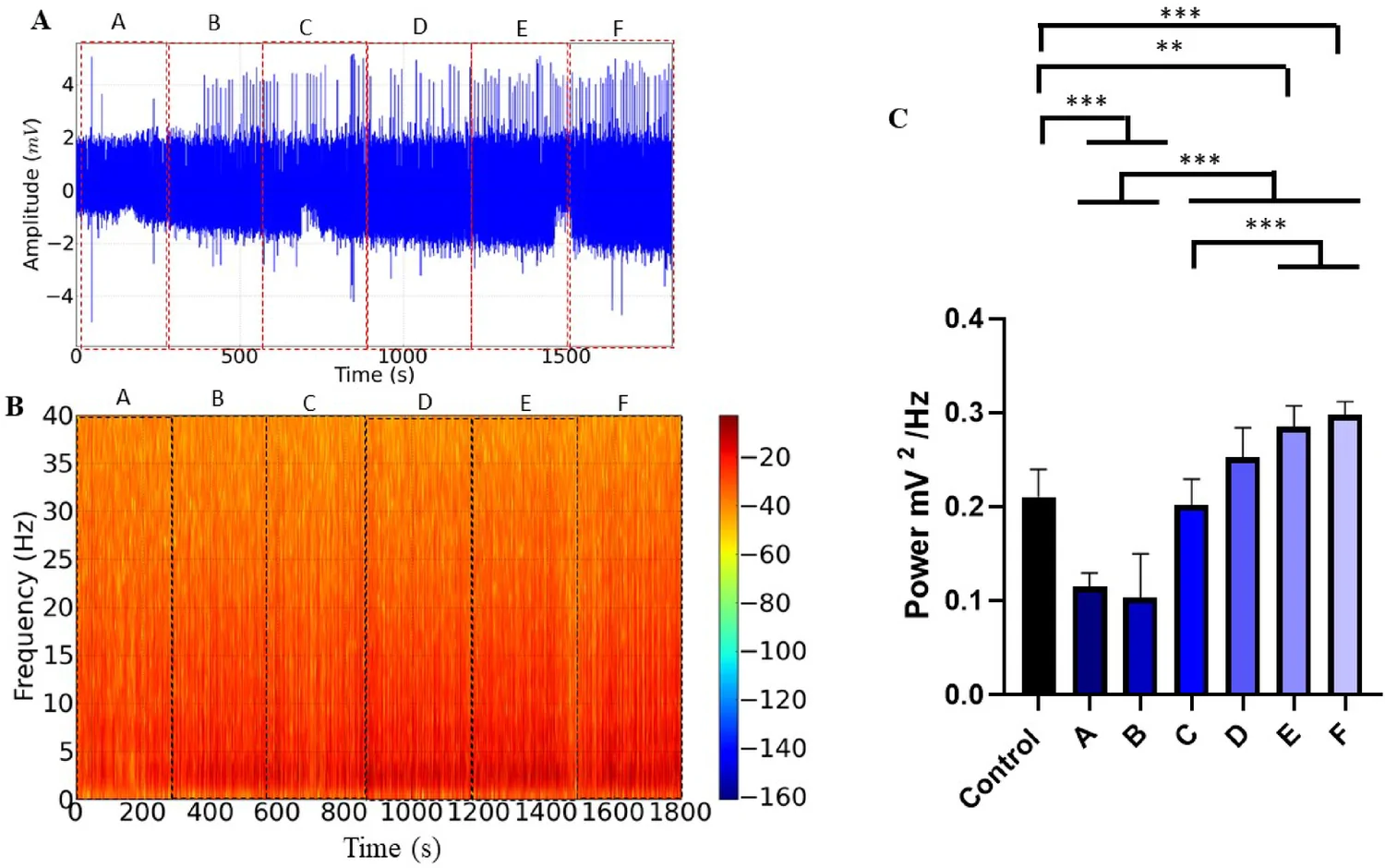
Electrocardiographic trace of *Anteos menippe* butterflies recorded over 30 min, showing the segments analyzed every 10 min (red dashed lines) (**A**), spectrogram of energy distribution illustrating regions with different energy intensities during restraint stress (**B**), Linear power graph of cardiac activity recorded during anesthetic induction and recovery (**C**) (after ANOVA applied by Tukey’s test; ***p < 0.001, *n* = 6)

Figure 3 shows the method of physical restraint applied to *Anteos menippe* butterflies for subsequent anesthetic induction for the group treated with isoflurane (A), the electrode insertion site for signal acquisition (B), and the characteristics of the electrodes manufactured for the experiment (C). Representative ECG recording showing the generation of impulses that maintain the heart rhythm (D), characterized by peaks with ascending and descending phases, as observed in the amplified recording (E).

The electrocardiogram illustrating the cardiac function of Anteos menippe butterflies in the untreated control group showed a mean peak heart rate of 61 ± 4.14 beats per minute (CV: 6.79%), with an amplitude of 2.46 ± 0.466 mV (CV: 18.93%)(signal amplification of 50,000×). The mean interval between peaks was 966 ± 86.79 ms (CV:8.67%). The contraction cycle responsible for driving the circulatory components, represented by the peak duration, had a mean value of 31.33 ± 3.26 ms (CV: 10.42%) (Fig. 2E).

ECG recordings of *Anteos menippe* butterflies during anesthetic induction and recovery with isoflurane, performed for 30 min, show variations in the amplitude of the graph element signal. From each recording, 5-minute segments were extracted for analysis in order to compare the effects of anesthesia on hemolymph pumping. During the first 5 min (0–300 s), low recording amplitude was observed, followed by a progressive increase in amplitude in the period of 300–600 s, while the amplitude remained stable during the periods of 600–900 s, 900–1200 s, 1200–1500 s and 1500–1800 s (Fig. 3A). Spectrogram analysis showed an increase in energy levels during the recovery periods starting from 300 to 1800 s, indicating changes in cardiac electrical activity during anesthetic recovery in the butterfly’s hemodynamics (Fig. 3B).

During the 30-minute cardiac recording, differences in the mean linear power of the signals were observed. In the 0–300 s period, the mean linear power was 0.114 ± 0.015 mV²/Hz × 10⁻³, like the 300–600 s period (*p* = 0.993), but lower than in the other groups. The control group was 0.210 ± 0.029 mV²/Hz × 10⁻³ was similar to periods C (*p* = 0.998) and D (*p* = 0.166), but was lower than group E and F. Periods C (0.201 ± 0.027 mV²/Hz × 10⁻³) was similar to period D (*p* = 0.0571), but was lower than period E and F. Periods D (0.252 ± 0.031 mV²/Hz × 10⁻³) E and F were similar (*p* = 0.119)(3 C), demonstrating that in the anesthetic recovery period there was a progressive increase in signal power throughout the recording.

**Fig. 4:**
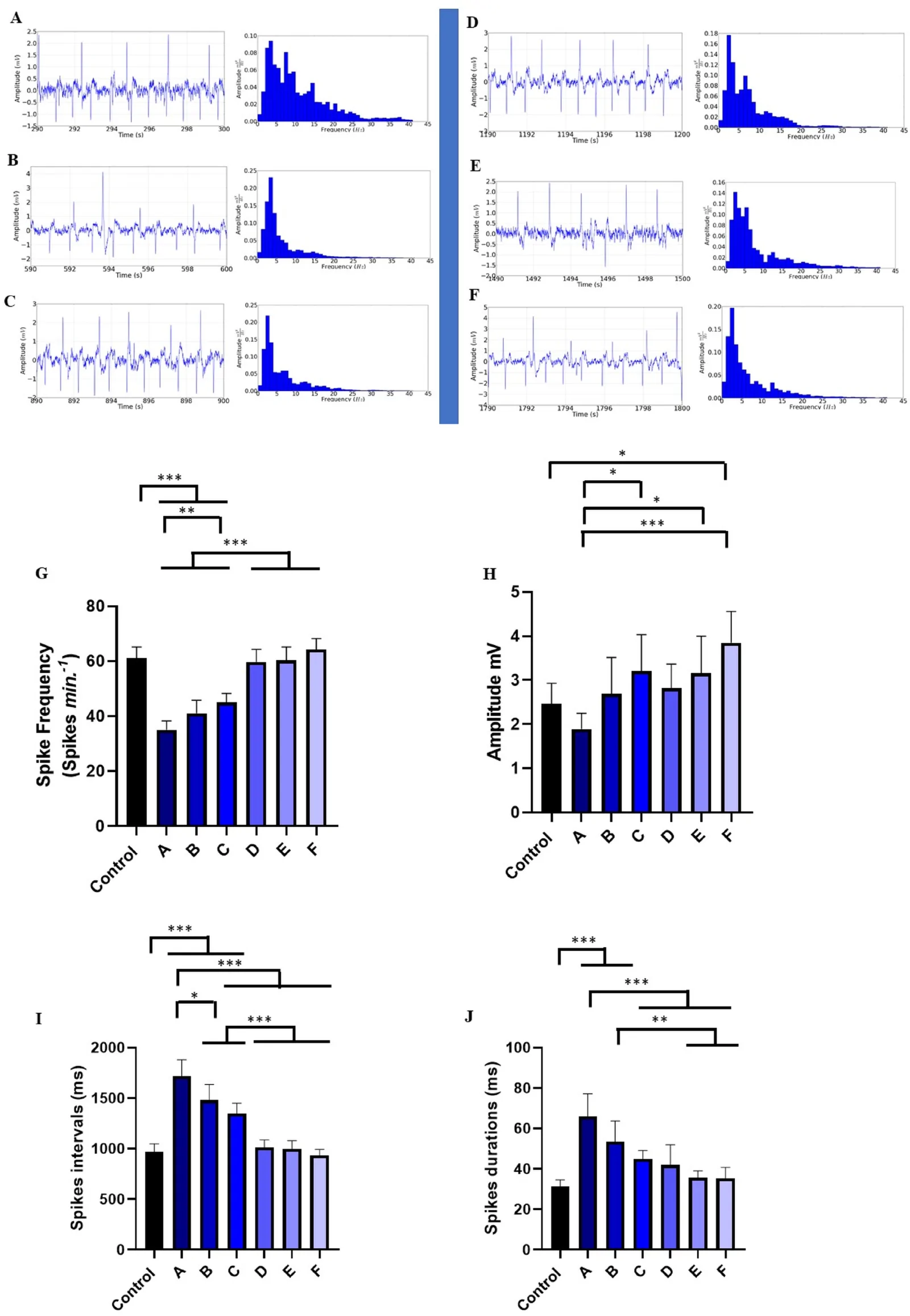
ECG tracing patterns recorded over 30 min, analyzed in 5-min segments, with corresponding power distribution histograms up to 40 Hz: 0–300 s (**A**), 300–600 s (**B**), 600–900 s (**C**), 900–1200 s (**D**), 1200–1500 s (**E**) and 1500–1800 s (**F**). Graphs show mean spike frequency per minute (**G**), spike amplitude (mV) (**H**), interspike interval (ms) (**I**), and spike duration (ms) (**J**). Data were analyzed using ANOVA followed by Tukey’s test. *p < 0.05, **p < 0.01, ***p < 0.001 (*n* = 6)

Each change in the ECG tracings during anesthetic induction and recovery in periods A, B, C, D, E, and F was analyzed as a pattern of cardiac activity (Fig. 4A–F). For period A, the mean peak frequency was 35 ± 3.28 per minute, with well-defined peaks showing positive deflections, and the histogram indicated the highest concentration of energy between 3 and 4 Hz with good distribution up to 25 Hz (Fig. 4A). In interval B, a change in peak direction was observed, with negative deflections predominating; the peak frequency increased to 41.0 ± 4.85 per minute, and the histogram showed maximum power at 4 Hz (Fig. 4B). In period C (600–900 s), maintenance in signal amplitude was observed, with a peak frequency of 45.0 ± 3.2 per minute, exhibiting positive and negative deflections, and the histogram showed peak power at 3 Hz (Fig. 4C). During period D (900 to 1200 s), the peak frequency was 59.67 ± 4.63 per minute, with positive and negative peaks and a predominance of 3 Hz in the histogram (Fig. 4D). For period E (1200–1500 s) the frequency was 60.33 ± 4.8 per minute, with a predominance in the frequencies of 3 to 6 Hz (Fig. 4E) and Period F (1500–1800 s) presented an average peak frequency of 64.33 ± 3.88 per minute with a predominance in the frequency range between 2 and 4 Hz (Figure F).

The frequency of peaks varied throughout the anesthetic induction and recovery periods. The control group presented a mean frequency of 61.0 ± 4.14 per minute, which was like periods D (*p* = 0.997), E (*p* = 0.999), and F (*p* = 0.806), but was higher than periods A, B, and C. Period A (35 ± 3.2 per minute) was like period B (*p* = 0.194). Period B (41 ± 4.8 per minute) was like period C (*p* = 0.646) but was lower than the subsequent periods. Periods D, E, and F were similar (*p* = 0.471) (Fig. 4G).

The signal amplitude increased throughout recovery. For the control group, the mean amplitude was 2.46 ± 0.46 mV, like periods A (*p* = 0.762), B (*p* = 0.996), C (*p* = 0.482), D (*p* = 0.969) and E (*p* = 0.563). However, it was lower than period F. Period A (1.89 ± 0.35 mV) was similar to periods B (*p* = 0.400) and D (*p* = 0.240), however, it was lower than the other periods (Fig. 4H).

For the inter-peak intervals, the control presented a mean of 963 ± 83.79 ms, which was lower than periods A, B and C, but was like periods D (*p* = 0.992), E (*p* = 0.999) and F (*p* = 0.998). Period A (1718 ± 163.8 ms) was higher than all other intervals. Period B (1479 ± 158.8 ms) was similar to period C (*p* = 0.411). Period D (1010 ± 77.62 ms) was like periods E (*p* = 0.999) and F (*p* = 0.898) (Fig. 4I).

The peak duration increased during the anesthesia period. The control had a mean peak duration of 31.33 ± 3.26 ms, which was lower than periods A and B, but was like groups C (*p* = 0.0512), D (*p* = 0.231), E (*p* = 0.953), and F (*p* = 0.968). Periods A (66 ± 11.22 ms) were like period B (*p* = 0.086) but were higher than the other periods. Period B (53.33 ± 10.39 ms) was like periods C (*p* = 0.4938) and D (*p* = 0.1503). Periods C, D, E, and F were similar (*p* = 0.319) (Fig. 4J).

## Discussion

Our study was pioneering in demonstrating heart rate parameters in butterflies of the species *Anteos menippe*, as well as investigating the effects of the volatile anesthetic isoflurane during anesthetic induction and recovery, using electrophysiology as a tool and demonstrating changes in electrocardiographic functionality during the procedure. The anesthetic produced the expected results, inducing sedation with gradual deepening, as evidenced by the loss and subsequent recovery of the postural reflex. The butterflies did not exhibit behaviors indicative of excitability caused by the anesthetic, demonstrating a positive result with gradual and gentle induction. The reduction in spike frequency observed during anesthetic induction likely reflects transient depression of neural and cardiac excitability mediated by GABAergic modulation induced by isoflurane. Similar chronotropic suppression has been described in other invertebrates exposed to volatile anesthetics, suggesting a conserved physiological response associated with reduced metabolic activity and neural signaling.

Although specific studies on butterfly species are lacking, isoflurane has a well-established use in invertebrate anesthesia and is considered superior to traditional methods such as cold exposure or CO₂ (MacMillan et al. 2017). Although cold exposure and CO₂ anesthesia remain widely used for insect immobilization, these methods may induce physiological disturbances such as metabolic suppression, ion imbalance, delayed recovery, and alterations in cardiac rhythmicity (Nilson et al. 2006; Gooley and Gooley 2021). In butterflies, which generally exhibit lower metabolic demands than highly active eusocial insects, these effects may differ in magnitude; however, the direct electrophysiological consequences of these methods remain poorly understood. In this context, the rapid induction and recovery promoted by isoflurane may represent important advantages for physiological investigations requiring stable cardiac monitoring. Its sedative efficacy has been reported in other species, including button-wing mantises (*Parasphendale agrionina*), emperor scorpions (*Pandinus imperator*), and yellow uruçu bees (*Melipona flavolineata*), showing mild induction and positive prognosis without observable adverse effects, consistent with our findings (D’Ovidio et al. 2021; Gaudette et al. 2022; Contrera et al. 2023). Therefore, isoflurane represents a highly effective and valuable anesthetic for invertebrates, with applications in scientific, commercial, and veterinary contexts.

The GABAergic system is widely conserved among insects and plays a central role in the modulation of neural excitability and motor activity. The electrophysiological parameters analyzed in this study provide important insights into cardiac regulation in butterflies. The frequency of spikes reflects chronotropic activity and may indicate alterations in autonomic regulation and, consequently, indirectly in metabolic demand, due to anesthetic modulation of cellular excitability. Interspike interval is inversely related to heart rate and may reflect alterations in pacemaker activity during anesthesia and recovery. Signal amplitude may be associated with the magnitude of electrical activity and the strength of myocardial contraction involved in hemolymph circulation involved in hemolymph circulation, whereas the duration of the spike may reflect changes in the temporal dynamics of cardiac electrical activity during each contraction cycle. Together, these parameters provide a physiological framework for evaluating stress responses and cardiac modulation in insects. In insects, GABA receptors are structurally and functionally well characterized, exhibiting conserved subunit composition, alternative splicing, and pharmacological sensitivity to several neuroactive compounds, including anesthetics and insecticides (Buckingham et al. 2005). Activation of this inhibitory system leads to neuronal hyperpolarization and reduced synaptic transmission, resulting in decreased motor output and behavioral quiescence. Such mechanisms provide a physiological basis for the sedative effects observed during anesthesia in insects and support the use of volatile anesthetics, such as isoflurane, as effective agents for inducing reversible immobilization without long-term adverse effects. Sedation is mediated by activation of the GABA system, leading to general inhibition of the central nervous system, resulting in reduced motor activity and loss of sensitivity to stimuli via mechanisms like those observed in vertebrates under anesthetic action. According to McClure and Heberlein (2013), these effects suggest that GABAergic modulation is an evolutionarily conserved pathway that regulates behaviors associated with torpor or deep rest, particularly during physiological processes such as diapause.

Studies in *Drosophila* have shown that GABAergic activity regulates the release of neuropeptides, such as corazonin (Crz), which influence reproductive cycle behaviors and rest states, including the control of diapause phases. These findings suggest that GABAergic modulation can impact physiological and behavioral processes associated with sedation, providing a conceptual basis for hypotheses regarding the physiological responses of *Anteos menippe* to sedatives.

This study represents the first direct electrocardiographic evaluation of butterflies, focusing on the effects of isoflurane during anesthetic induction and recovery in *Anteos menippe*. It is important to note that the peaks observed in insects are not functional equivalents of the human QRS complex, although they can be used for physiological analogies. Our findings are consistent with those reported by Davis and Orakzai (2016), who documented mean resting heart rates of 63 bpm in butterflies, supporting the physiological congruence between lepidopterans from different biomes. Furthermore, the characterization of electrophysiological phases in butterflies aligns with observations in other arthropods, as reported by Sláma (2012), revealing homologous patterns to the cardiac complexes of arthropods when compared to vertebrates and reinforcing the evolutionary conservation of autonomic nervous system mechanisms in cardiac contraction. Methodologically, our study complements the approach proposed by Wang and Chahl (2025), highlighting the value of high-resolution electrophysiological monitoring to ensure animal welfare and detailed spectral analysis.

Regarding the spectrographic analysis, the increase in spike frequency over time, associated with postural recovery after anesthesia, suggests a physiological behavior similar to that observed in bees by Contrera et al. (2023), indicating a shortening of the interspike interval during the recovery period. The progressive increase in signal amplitude and linear power during recovery may indicate restoration of cardiac electrical activity and hemolymph propulsion dynamics following anesthetic elimination.

Furthermore, oscillations in amplitude and energy over time may reflect changes in cardiac activity after anesthetic removal, indicating alterations in cardiac electrical conduction and hemodynamic effects. Overall, these results demonstrate that the cardiac activity of *A. menippe* follows universal patterns of chronotropic variability described in insects, while also extending the physiological framework to a broader range of species. The establishment of electrophysiological monitoring protocols in butterflies may also contribute to ecotoxicological studies investigating the effects of pesticides, pollutants, environmental stressors, and climate-related disturbances on insect physiology and survival.

## Conclusion

The results of this study demonstrate, for the first time, that isoflurane is an effective and safe anesthetic agent for managing *Anteos menippe* butterflies, providing gradual induction, predictable recovery, and the absence of undesirable excitatory effects. Electrocardiographic characterization performed during anesthetic induction and recovery revealed physiological patterns consistent with autonomic modulation previously described in other insects, demonstrating that the cardiac system of this species follows conserved mechanisms of chronotropic variability. The progression of electrical responses, reflected in changes in the frequency, amplitude, and energy distribution of the recorded peaks, reinforces the applicability of the ECG as a monitoring tool in invertebrates, expanding the possibilities for investigating physiological parameters in experimental and conservation contexts. Thus, this work not only contributes to the refinement of anesthetic practices in lepidopterans but also establishes a relevant methodological basis for future studies aimed at understanding cardiac physiology in insects, strengthening the scientific framework necessary for the preservation and well-being of these organisms in laboratory and management environments.

## Data Availability

All data generated or analyzed during this study are available in the public repository at the following link: [https://drive.google.com/file/d/1tXFolmPTa7sO4esOJahb4U2E6W1DuXz_/view? usp=sharing](https:/drive.google.com/file/d/1tXFolmPTa7sO4esOJahb4U2E6W1DuXz_/view? usp=sharing). The datasets support the findings of this study and include the original electrophysiological and behavioral data.
